# Intracameral Interleukin 1β, 6, 8, 10, 12p, Tumor Necrosis Factor α and Vascular Endothelial Growth Factor and Axial Length in Patients with Cataract

**DOI:** 10.1371/journal.pone.0117777

**Published:** 2015-02-13

**Authors:** Dan Zhu, Da-Yong Yang, Yuan-Yuan Guo, Yan-Fei Zheng, Jun-Lian Li, Bin Wang, Yong Tao, Jost B. Jonas

**Affiliations:** 1 Department of Ophthalmology, the Affiliated Hospital of Inner Mongolia Medical University, Hohhot, Inner Mongolia, China; 2 Department of Ophthalmology, People’s Hospital, Peking University, Beijing, China; 3 Department of Ophthalmology, Medical Faculty Mannheim of the Ruprecht-Karls-University Heidelberg, Seegartenklinik Heidelberg, Germany; University Zürich, SWITZERLAND

## Abstract

**Objective:**

To assess associations between the aqueous humour concentration of interleukin IL-1β, IL-6, IL-8, IL-10 and IL-12p, tumor necrosis factor α (TNF-α) and vascular endothelial growth factor (VEGF) and axial length in eyes with cataract.

**Methods:**

The hospital-based investigation included patients who underwent cataract surgery between March 2014 and April 2014. Using aqueous humour collected at the start of cataract surgery, the interleukins IL-1β, IL-6, IL-8, IL-10 and IL-12p, TNF-α and VEGF were examined using a cytometric bead array. Axial length was determined by partial coherence laser interferometry (IOL Master).

**Results:**

The study included 33 patients with cataract (33 eyes) with a mean age of 69.2±10.8 years (range:50–87 years) and a mean axial length of 24.7±1.9 mm (range:22.6–31.5 mm). Lower aqueous concentration of VEGF was significantly associated with longer axial length (VEGF concentration (pg/mL) = -5.12 x Axial Length (mm) + 163; correlation coefficient r = -0.41; P<0.001) and more myopic refractive error (VEGF concentration (pg/mL) = 1.27xspherical equivalent (diopters)+44.8; r = 0.383; P = 0.002). The aqueous concentrations of all other substances were not significantly (all P>0.10) associated with axial length or refractive error.

**Conclusions:**

Higher intravitreal concentrations of VEGF were measured in eyes with a longer axial length, while the intraocular concentrations of IL-1β, IL-6, IL-8, IL-10, IL-12p and TNF-α were not correlated with axial length. The lower concentration of VEGF in axially elongated eyes may be one of the reasons for the lower prevalence of age-related macular degeneration and diabetic retinopathy in myopic eyes.

## Introduction

Cytokines are of utmost importance for physiology and pathophysiology of the retina and retinal disorders. Under physiological conditions, vascular endothelial growth factor (VEGF) is secreted by several intraocular cell types such as the retinal pigment epithelium cell to maintain the fenestrated structure of the endothelium of the choriocapillaris and for other functions. Under pathophysiological conditions, VEGF has been shown to be strongly related with intraocular neovascularization in various diseases including exudative age-related macular degeneration, diabetic retinopathy and retinal vein occlusions [1–4]. Other cytokines such as interleukins and tumor necrosis factor α (TNF-α) are strongly associated with inflammatory disorders in the eyes, including diseases such as diabetic retinopathy with diabetic macular edema [5–7]. Previous population-based studies in various world regions revealed that, in multivariate analysis, myopic eyes versus hyperopic eyes had a lower prevalence of age-related macular degeneration and diabetic retinopathy [8–17]. Since the severity of these diseases, and in particular their complications, is related to the intraocular concentration of these cytokines [18,19], and since the concentration of a substance depends on the available volume, in which the substance is distributed, we conducted this study to assess whether the physiological intraocular concentrations of these cytokines were related to the size of the globe.

## Methods

The hospital-based investigation included patients who underwent cataract surgery between March 2014 and April 2014. The ethics committee of the Affiliated Hospital of Inner Mongolia Medical University approved the study and all study participants gave their informed written consent. Exclusion criteria were any previous intraocular surgery, glaucoma, any retinal or vitreoretinal disorder such as diabetic retinopathy, retinal vein occlusion and age-related macular degeneration, and systemic diseases such as diabetes mellitus and rheumatic disorders. If patients underwent cataract surgery in both eyes in the study period, only the eye operated first was included into the study.

All patients underwent an ophthalmologic examination including refractometry (auto-refractometer ARK-900, NIDEK, Tokyo, Japan), tonometry and slit lamp assisted biomicroscopy of the anterior segment and posterior segment of the eye. Additionally, biometry (IOL Master; Version 3.01; Carl Zeiss Meditec AG, Germany) was performed to measure axial length. We additionally calculated the intraocular volume by assuming the eye globe having a spherical shape and applying the formula for calculation of a sphere´s volume. The same procedure had previously been applied by Teichmann, and by Meyer and colleagues [20,21].

Aqueous humour was collected at the start of cataract surgery. After disinfection of the periorbital skin and conjunctiva, sterile draping of the patient, and insertion of a lid speculum, a paracentesis was performed in the temporal limbal region. An aqueous humor sample of a volume of at least 100 μL was aspirated through a 26 gauge syringe. The samples were immediately deeply frozen in liquid nitrogen. The concentration of interleukin 1β, 6, 8, 10, 12p (IL-1β, IL-6, IL-8, IL-10, IL-12p), TNF-α and vascular endothelial growth factor (VEGF) were measured by cytometric bead array which is a method of capturing a soluble analyte or set of analytes with beads of known size and fluorescence, making it possible to detect analytes using flow cytometry. The detailed technique has been described previously [22,23]. The selection of the cytokines examined in the study was based on their potential importance in the intraocular neovascular or edematous diseases and on the availability of examination kits to measure all them together in a single aqueous humour sample.

Statistical analysis was performed using SPSS for Windows, version 21.0 (IBM-SPSS, Chicago, Illinois, USA). The refractive data were converted into the spherical equivalent for statistical analysis. The Kolmogorov-Smirnov test was applied to test for a normal distribution of the parameter. Kendall correlation test was used to evaluate the bivariate correlation. Two-tailed probabilities of less than 0.05 were considered to indicate statistical significance. Confidence intervals (95%CI) were calculated.

## Results

The study included 33 patients (33 eyes, 12 women) who underwent routine cataract surgery. The mean age of patients was 69.2 ± 10.8 years (range, 50 to 87 years), mean intraocular pressure was 15.7 ± 3.6 mmHg (range, 10 to 21 mmHg), and mean axial length was 24.7 ± 1.9 mm (range: 22.6–31.5 mm).

Mean concentration of VEGF was 36.6 ± 19.2 pg/mL, of TNF-α it was 3.95 ± 0.69 pg/mL, of IL-1ß it was 5.29 ± 0.9 pg/mL, of IL-6 it was 68.3 ± 160.8 pg/mL, of IL-8 it was 12.1 ± 4.1 pg/mL, of IL-10 it was 4.46 ± 0.62 pg/mL, and of IL-12p70, it was 0.89 ± 1.56 pg/mL.

The Kolmogorov-Smirnov test showed that the concentrations of all cytokines including IL-1β, IL-6, IL-8, IL-10, IL-12p, TNF-α and VEGF did not fit a normal distribution curve, so that Kendall correlation test was used to evaluate the bivariate correlation between axial length or spherical equivalent and the aqueous level of varied cytokines.

**Fig 1:**
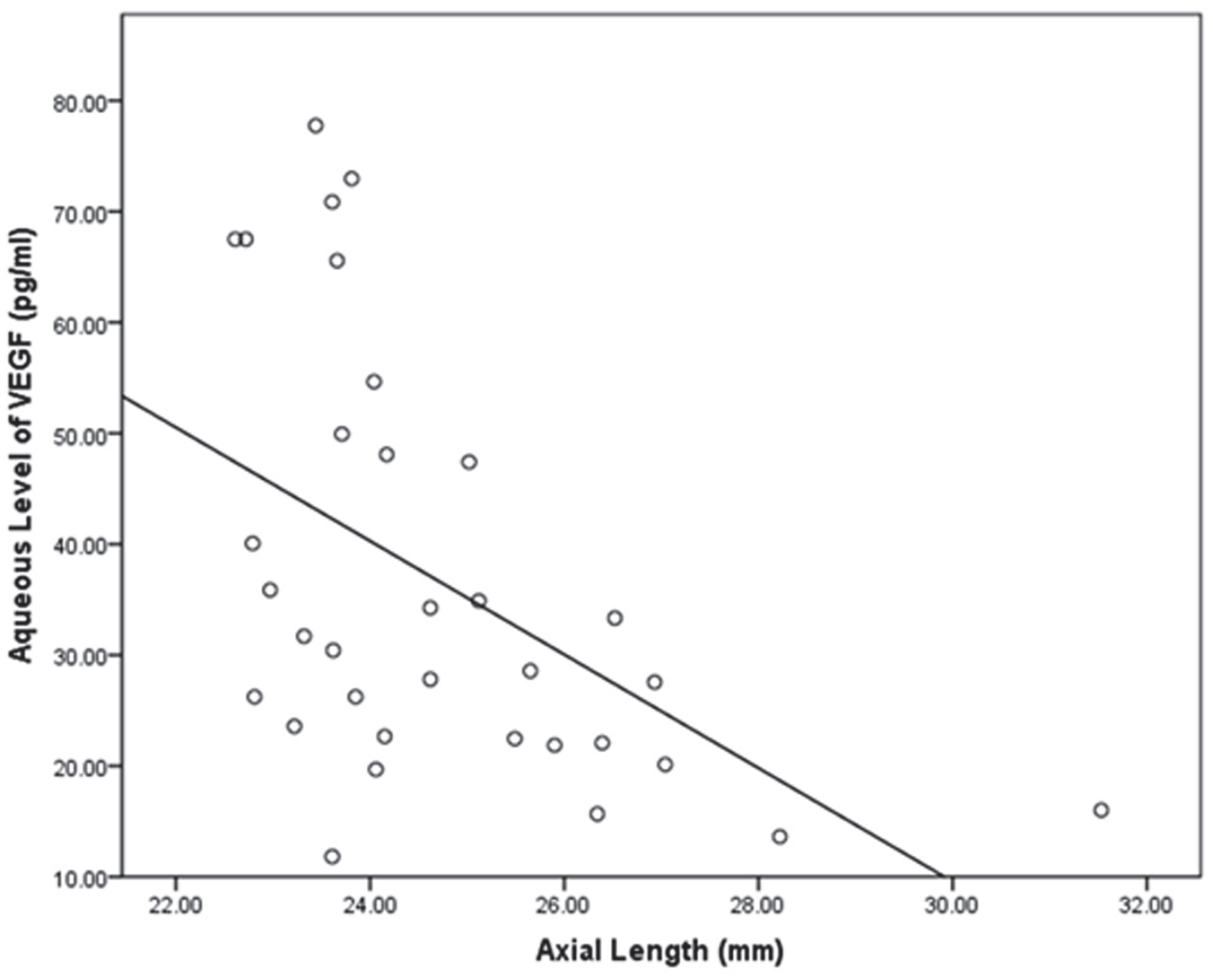
Scattergrams showing the associations between axial length and the concentrations of vascular endothelial growth factor (VEGF) in the aqueous humour of patients without retinal diseases and undergoing routine cataract surgery.

Lower aqueous concentrations of VEGF were significantly (*P* = 0.003; standardized correlation coefficient beta: 0.50) associated both with longer axial length (equation of the regression line: VEGF concentration (pg/mL) = -5.12 x Axial Length (mm) + 163, r = -0.41) (Fig. 1). In addition, higher aqueous humour concentration of VEGF were significantly associated with older age (r = 0.30; *P* = 0.02).

In a multivariate analysis, with VEGF concentration as dependent variable and age and axial length as independent variables, lower VEGF concentrations were significantly associated with longer axial length (*P* = 0.016; beta: -0.39; correlation coefficient B: -3.98; 95%CI: -7.14, -0.81) and older age (*P* = 0.026; beta: 0.36; B: 0.64; 95%CI: 0.90, 1.11)

If instead of axial length the calculated ocular volume was taken as independent variable, the standardized correlation coefficients were similar (beta: -0.50 for axial length; beta: -0.48 for ocular volume).

**Fig 2:**
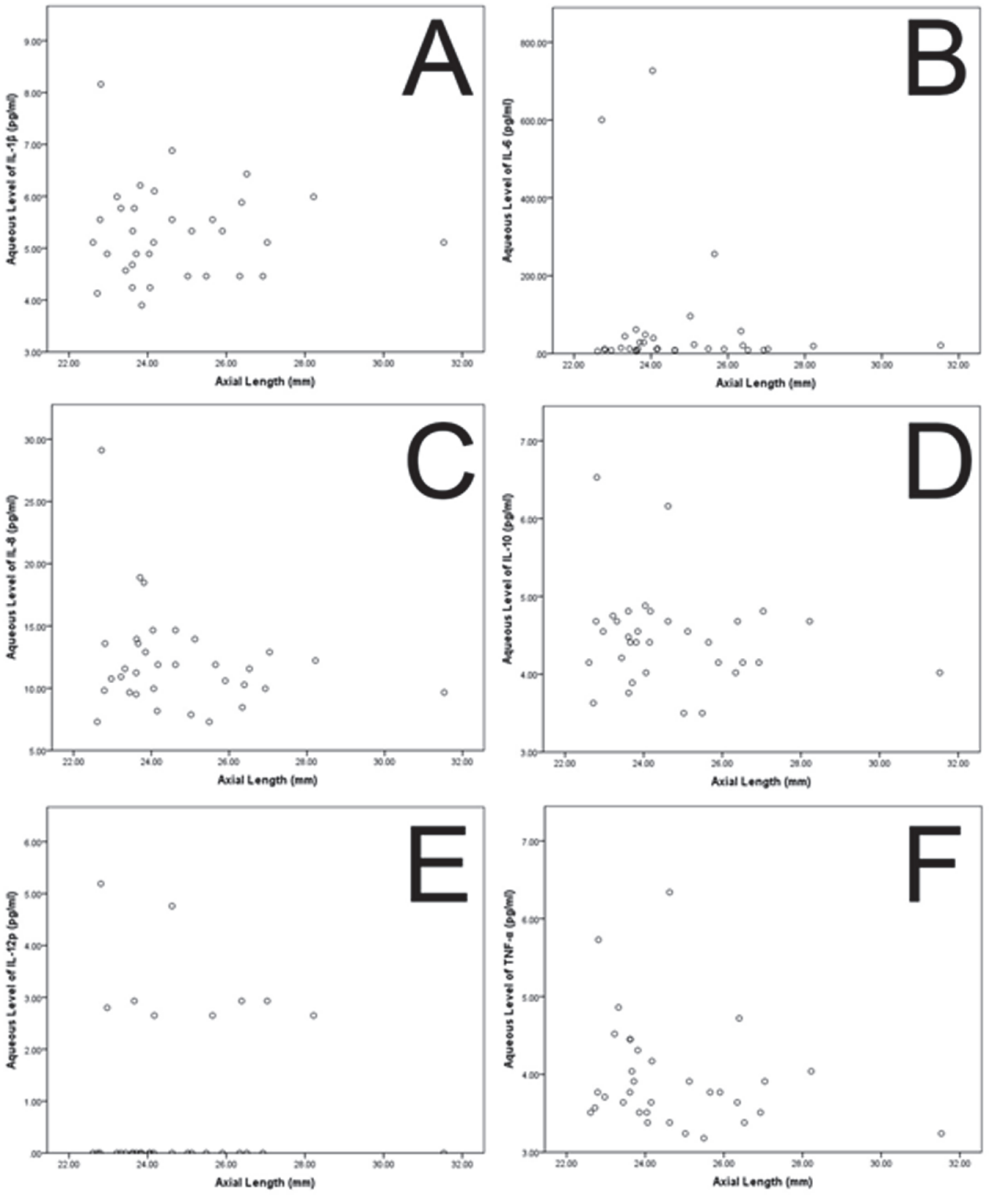
Scattergrams showing the associations between axial length and the concentrations of interleukin 1β, 6, 8, 10, 12p (IL-1β, IL-6, IL-8, IL-10, IL-12p), tumor necrosis factor α (TNF-α) (A-F) in the aqueous humour of patients without retinal diseases and undergoing routine cataract surgery.

**Fig 3:**
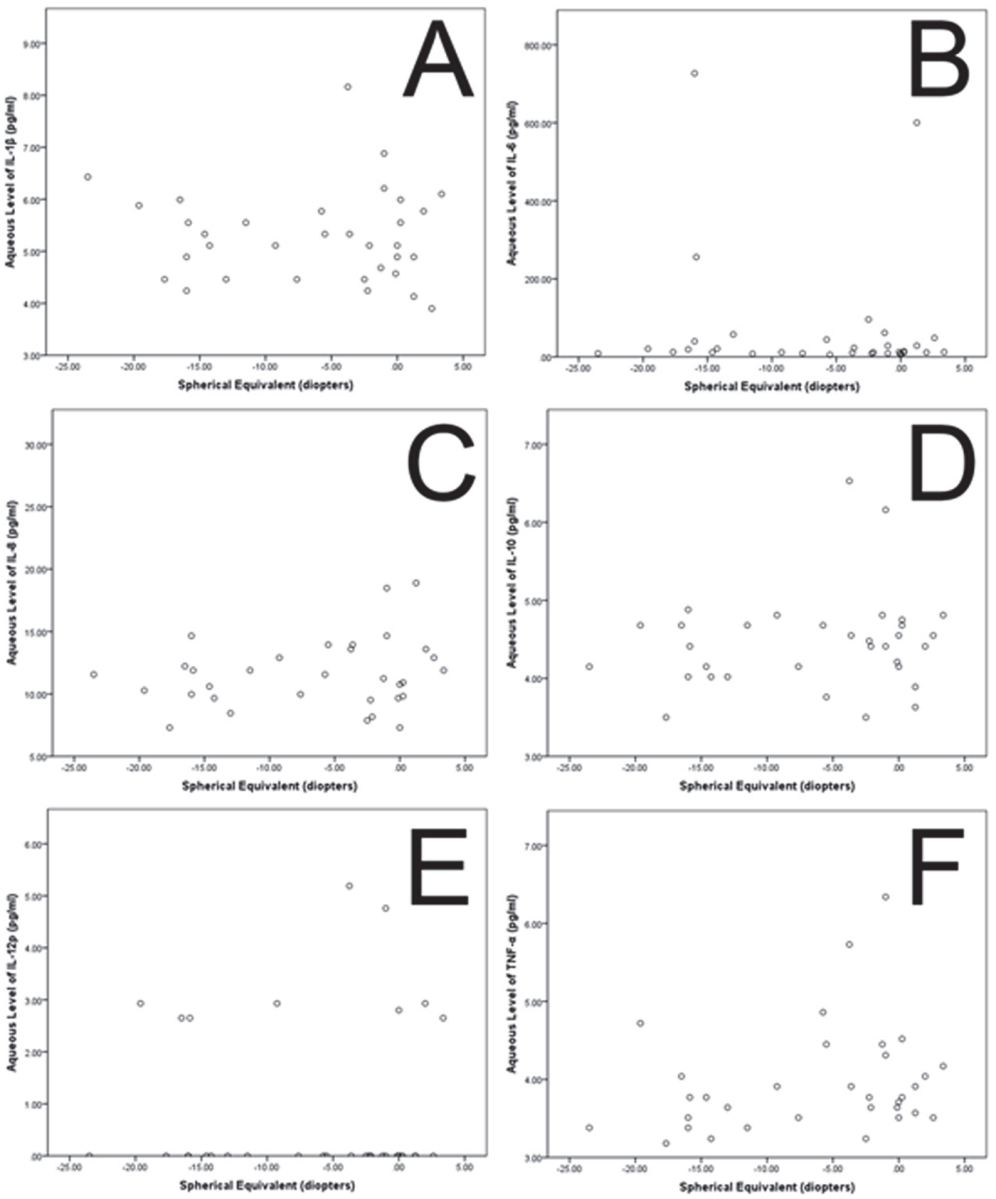
Scattergrams showing the associations between refractive error (spherical equivalent) and the concentrations of interleukin 1β, 6, 8, 10, 12p (IL-1β, IL-6, IL-8, IL-10, IL-12p), tumor necrosis factor α (TNF-α) (A-F) in the aqueous humour of patients without retinal diseases and undergoing routine cataract surgery.

The concentrations of all other substances tested were significantly associated neither with axial length (all *P*>0.1) or spherical equivalent (all *P*>0.1) (Fig. 2, 3) or with age or gender (all *P*>0.05).

## Discussion

The results of our study again confirmed that intracameral concentrations of VEGF was significantly associated with axial length and spherical equivalent, with a larger number of people who belong to another racial group. However, the association was not present between inflammation cytokines including IL-1β, IL-6, IL-8, IL-10, IL-12p, TNF-α and axial length or spherical equivalent.

The aqueous humour concentrations of VEGF as determined in our study (36.6 ± 19.2 pg/mL) agreed with the results of a previous studies with a similar design, in which intracameral VEGF concentrations of 38 ± 39 pg/mL were measured [24]. In eyes with retinal or choroidal disorders, markedly higher concentration were determined such as 50 pg/mL (median) in eyes with uveal malignant melanomas [25], 108 ± 72 pg/mL in eyes with polypoidal choroidal vasculopathy and choroidal neovascularization [26], 351 ± 273 pg/mL in eyes with branch retinal vein occlusion [27], and 337 ± 117 pg/mL in eyes with retinitis pigmentosa [28].

In our study, the intraocular concentration of VEGF decreased significantly with increasing axial length, and as a corollary, with increasing myopic refractive error. It is in agreement with previous investigations on individuals of other ethnicity in whom in a similar manner the intracameral concentration decreased with increasing axial length [24, 29–31]. It is also in agreement with a recent investigation with similar results on the intravitreal concentration of VEGF decreasing with longer axial length [32]. Since axial length is related to ocular volume and assuming that hyperopic eyes and myopic eyes do not differ in the physiologic production rate of VEGF, one may infer that the lower intraocular concentrations of VEGF in the axially elongated myopic eyes were due to a more marked dilution of VEGF in the myopic eyes. Since VEGF plays an important role in the in the pathogenesis of intraocular neovascular diseases such as exudative age-related macular degeneration and proliferative diabetic retinopathy, one may assume that the physiologically lower intraocular concentration of VEGF in myopic eyes versus hyperopic eyes may be one of the reasons for the lower prevalence of age-related macular degeneration and diabetic retinopathy in myopic eyes [8–17]. Other reasons, besides a larger volume leading to dilution, for the lower VEGF concentration in axially long eyes may be differences in the consistency of the vitreous, which is less viscous in axially elongated eyes. A higher fluidity of the vitreous may be associated with a faster turnover of VEGF out of the eye.

Interestingly, Krohne and colleagues did not find a significant correlation between the aqueous humour concentration of intravitreally injected ranibizumab or bevacizumab and axial length [33]. They assessed the effects of ocular axial length on pharmacokinetics and duration of action of the VEGF inhibitors ranibizumab and bevacizumab after intravitreal injection in patients. In 119 patients, aqueous humor was sampled at different time points after the intravitreal application of the drug and the relative deviation of the measured drug concentrations from the time-corrected mean values was calculated for 41 eyes. Krohne and coworkers found for neither ranibizumab nor bevacizumab a correlation between ocular pharmacokinetics (as measured by relative deviation of drug concentration from the mean) and axial length. Future studies may address why the intraocular concentration of intravitreally applied drugs appeared to be statistically independent of axial length and ocular volume, while the intraocular concentration of VEGF decreased with longer axial length (Fig. 1).

In our study, we additionally measured the aqueous humour concentration of other, pro-inflammatory and inflammatory, cytokines such as IL-1β, IL-6, IL-8, IL-10, IL-12p and TNF-α and thus extended the findings of previous studies which were focused on the intraocular concentration of VEGF [24, 29–31]. IL-1 and TNF-α increase the expression of adhesion molecules on both endothelial cells and leukocytes, and promote leukocyte infiltration from the blood into inflamed tissue. IL-1β induces morphological transformation in human dermal micro-vascular endothelial cells, accompanied by an increased growth rate, loss of contact inhibition, and an increase in the permeability of confluent endothelial cell monolayers. Both IL-1β and VEGF induce each other and are both essential to induce an angiogenic response, and neutralization of either of these cytokines abrogated the angiogenic response. IL-6 is a pleiotropic cytokine that regulates multiple biological processes, including the development of the nervous and hematopoietic systems, acute-phase responses, inflammation, and immune responses. As an important pro-inflammatory cytokine, it is derived from activated T-lymphocytes with multiple functions, including induction of B-cell differentiation and antibody production, induction of B-cell growth, induction of differentiation and proliferation of T cells, and induction of hepatocyte secretion of acute-phase inflammatory proteins. IL-8 is associated with the recruitment of monocytes and neutrophils, the signature cells of acute inflammatory response. Cellular recruitment occurs through the development of a chemotactic gradient, which causes the inflammatory cell to move towards an area of increased chemokine concentration. This gradient aids in bringing cells towards the site of inflammation and also retains them once they have arrived. The expression of these pro-inflammatory or inflammatory cytokines were reported to be increased in a variety of ocular pathological disorders, such as diabetic macular edema and exudative age-related macular degeneration [34–37]. Furthermore, it has been reported that vitreous concentrations of IL-1, IL-6, and TNF-α were increased in an experimental uveitis rat model [38].

Interestingly, the intracameral concentration of these pro-inflammatory and inflammatory cytokines (IL-1β, IL-6, IL-8, IL-10, IL-12p, TNF-α) were not significantly associated with axial length in our study (Fig. 1). It is in agreement with a previous study in which the intracameral concentrations of three other inflammatory cytokines, MCP-1, sICAM-1 and sVCAM-1 were neither associated with refractive error nor with axial length [18]. In the same study, the intracameral VEGF concentrations decreased with longer axial length. It has remained unclear why in three independent studies, the intraocular concentrations of VEGF showed an inverse relationship with axial length while this association was not valid for the intracameral concentration of other cytokines. One of the reasons could be that the physiological concentration of VEGF is markedly higher than the physiological concentrations of most other cytokines, so that the accuracy of the measurement method might not have been sufficient to detect minor differences and associations. Another explanation for the lack of a detected association between the concentrations of interleukins and of TNF-α with axial length may have been that these cytokines may primarily not be produced in the eye. If they leak into the eye, their intraocular concentration may depend on the inner ocular surface which increases with axial length. In that case the diluting effect of an increasing axial length may have been compensated.

The finding of our and previous studies on a lack of an association between the intraocular concentration of interleukins and TNF-α and axial length contrasts with the finding of an investigation by Jia and colleagues who reported that the aqueous humor concentrations of matrix metalloproteinase 2 (MMP-2) and of tissue inhibitors of matrix metalloproteinases (TIMP) TIMP-1, TIMP-2 and TIMP-3 were elevated in eyes with elongated axis [39].

Limitations of our study should be mentioned. Firstly, the number of the patients enrolled was relatively low. Despite of it, the results for the inverse association between the intracameral concentration of VEGF and axial length were statistically significant. The relatively small number of patients may thus serve to strengthen the conclusions on that association. Second, the relatively small number of individuals included into the study may not have provided a sufficient statistical power to prove an association between axial length and the intracameral concentration of the other cytokines tested.

In conclusion, higher intravitreal concentrations of VEGF were measured in eyes with a longer axial length, while the intraocular concentrations of IL-1β, IL-6, IL-8, IL-10, IL-12p and TNF-α were not correlated with axial length. The lower concentration of VEGF in axially elongated eyes may be one of the reasons for the lower prevalence of age-related macular degeneration and diabetic retinopathy in myopic eyes.
